# Segmental analysis of human hair reveals intra-annual variation in 25(OH)D_3_ concentrations in modern and archaeological individuals

**DOI:** 10.1038/s41598-025-86097-6

**Published:** 2025-01-24

**Authors:** Kate Britton, Orsolya Czére, Eléa Gutierrez, Linda M. Reynard, Eamon Laird, Gary Duncan, Baukje de Roos

**Affiliations:** 1 Department of Archaeology, University of Aberdeen, Elphinstone Road, Aberdeen, UK; 2 AASPE “Archéozoologie, Archéobotanique: Sociétés, Pratiques, Environnements”, Muséum National d’Histoire Naturelle, 75005 Paris, France; 3 Department of Geosciences, Boise State University, Boise, ID USA; 4 Department of Health & Nutritional Sciences, Atlantic Technological University (ATU), Sligo, Ireland; 5 The Rowett Institute, University of Aberdeen, Foresterhill, Aberdeen, UK

**Keywords:** Vitamin D, 25-hydroxyvitamin D3, Metabolomics, Stable isotopes, Palaeopathology, Palaeodiet, Metabolomics, Mass spectrometry, Archaeology

## Abstract

Vitamin D is essential for healthy skeletal growth and is increasingly recognised for its role in chronic disease development, inflammation and immunity. 25-hydroxyvitamin D_3_ (25(OH)D_3_) concentrations are an indicator of vitamin D status and are normally analysed in plasma or serum samples in clinical settings, while archaeological studies rely on the identification of skeletal markers of vitamin D deficiency, such as rickets. Here, we determined 25(OH)D_3_ concentrations in hair specimens (‘locks’) that had been sampled close to the root, aligned by cut end, and sliced into sequential segments from participants (*n* = 16), from Aberdeen, Scotland, using a modified protocol designed to minimise sample size. Concentrations were above detectable levels in 14 of 16 individuals, generating a (~ monthly) time-series of 25(OH)D_3_ concentrations, with fluctuating intra-hair trends consistent with the bioaccumulation of 25(OH)D_3_. In three participants, fluctuations in intra-hair 25(OH)D_3_ appear linked to recent significant weight loss, potentially due to the release of stored 25(OH)D_3_ from adipose tissue and subsequent uptake in hair. For the remaining participants, no statistically-significant correlations were determined between mean hair 25(OH)D_3_ levels and self-reported data, including age, sex, BMI, vitamin D supplementation, frequency of oily fish consumption, and hours spent outside. For a subset of our cohort (*n* = 4) isotope analysis highlighted potential relationships between elevated *δ*^18^O values (which can indicate season of hair growth) and 25(OH)D_3_ concentrations in some individuals, which may reflect seasonally-increased UVB exposure. We also present data from an archaeological individual from the same city, with the addition of further isotope analysis (carbon, nitrogen, sulphur) to characterise diet. Results suggest possible positive correspondence of 25(OH)D_3_ levels with season in this archaeological individual, and possibly with marine protein consumption, highlighting the potential use of this approach in characterising the relationship between past vitamin D levels and diet. While results are promising, we recognise the limits of this study in terms of sample size and use of self-reported data, and further work is needed to better understand the relationship between serum and hair 25(OH)D_3_ before this approach can further be developed as either a non-invasive medical test or an archaeo-investigative technique.

## Introduction

Vitamin D is essential for healthy skeletal growth, and low vitamin D levels have been associated with a range of adverse health outcomes including inflammation, impaired immune function, osteoporosis and diabetes^1,2^. There are also interactions between vitamin D status and other physiological traits and conditions. For example, individuals living with obesity have a higher risk of vitamin D deficiency as vitamin D is a lipophilic molecule that can be stored in body fat rather than freely circulating in plasma^3^. Ultraviolet-B (UVB) radiation exposure is our primary source of vitamin D; and therefore, those residing at higher latitudes are at elevated risk of deficiency due to lower UVB exposure^4–6^. In such cases, dietary supply becomes essential. The consumption of vitamin D-rich foods, such as oily fish, has been associated with a reduced prevalence of low vitamin D levels over winter^7^ and many countries have public health recommendations and policies in place that promote the intake of vitamin D supplements, especially over winter, to prevent vitamin D deficiency^8,9^. Lack of availability (or consumption) of vitamin D-rich foods or pharmaceutical supplements however, can compound risk of deficiency and related health issues^10^.

The most common method of assessing vitamin D status in an individual is the measurement of serum or plasma 25(OH)D_3_ (also known as calcifediol or calcidiol) concentrations, which is a metabolite from the enzyme-mediated hydroxylation of vitamin D_3_ (cholecalciferol) in the liver. 25(OH)D_3_ concentration in serum or plasma is a biomarker that reflects both dietary intake and endogenous UVB skin production^11^. Although blood analysis is the traditional method, recent studies have demonstrated the potential to determine 25(OH)D_3_ levels in humans from cut samples of hair^12–14^ potentially offering a simpler, inexpensive and less invasive way to monitor vitamin D status than blood analysis. Importantly, this method also has the potential to contribute to advances in other disciplines. For example, in archaeology and palaeoanthropology, vitamin D status is normally implied through the identification of skeletal manifestations of deficiency (i.e., rickets or osteomalacia; analysis of dental defects; see review in^15^), but these approaches do not permit an empirical estimate of vitamin D status.

As with other recent developments in archaeometabolomics^16^, the direct measurement of 25(OH)D_3_ in tissues that can be found at archaeological sites, such as hair, would represent a significant methodological advancement for the field. It would also offer the potential to compare estimates of individual vitamin D status with other types of data. For example, stable isotope analysis (SIA) is commonly used in ecology and archaeology to estimate human and animal diets (for overviews and applications of SIA to ecology and archaeology, see^17–20^). Studies are based on the principle that the stable isotope ratios of biological tissues (such as those of hydrogen, oxygen, carbon, nitrogen and sulphur in the amino acids in keratin) are directly related to the isotopic ratios of food and water ingested, mediated by an individual’s physiology and metabolic processes. Applications are routinely underpinned by controlled feeding experiments, recruitment or field studies: for example, studies on human hair and fingernails have demonstrated that nitrogen isotope ratios (*δ*^15^N) reflect trophic level (e.g., ovo-lacto vegetarians and omnivores are progressively ^15^N-enriched relative to vegans, see^21–24^). Also, and due to the isotopically-distinct sources of carbon in marine and terrestrial ecosystems, carbon isotope ratios (*δ*^13^C) can (along with *δ*^15^N) indicate the consumption of marine foods^25,26^. The carbon (*δ*^13^C) and nitrogen (*δ*^15^N) SIA of archaeological hair can thus provide proxy evidence for the types of protein consumed during the period of hair growth (see a recent review by^27^ and the Supplementary Information) and, when paired with 25(OH)D_3_ data, could provide unique insights into the relationship between past diets and vitamin D health. Furthermore, and assuming an average growth rate of scalp hair of around ~ 1 cm per month in humans^28–30^, the cutting and analysis of longer locks of human hair as serial-sections could place such data within a time-series.

However, prior to the widespread application of 25(OH)D_3_ analysis to either modern or archaeological hair, further exploratory studies are needed. Sample sizes required are relatively large (20–50 mg, 50 mg; and 200 mg in^12–14^ respectively) and, while this is typical for the determination of endogenous steroids in hair^67^, it currently limits applications to finite archaeological materials, particularly where integration with other approaches (e.g., isotope analysis, aDNA analysis) may be desired. More fundamentally perhaps, although 25(OH)D_3_ can be measured in human hair and has even been shown to vary in consecutive samples taken down locks of hair from two different people^14^, the precise mechanism whereby 25(OH)D_3_ is incorporated into the tissue has not yet been established. 25(OH)D_3_, like cortisol, may enter the hair at the medulla via passive diffusion from blood. Alternatively, since the epidermis and its keratinocytes produce vitamin D_3_, all the enzymes required for vitamin D metabolism and vitamin D receptors are present in hair follicles; as a result, 25(OH)D_3_ could theoretically be produced in hair follicles and therefore be preserved in hair once grown^12,31^. The mode by which 25(OH)D_3_ is incorporated into hair, as well as its synthesis, absorption and circulation, influences the period of time a change in circulating serum 25(OH)D_3_ levels would be exhibited in growing hair and therefore the interpretation of intra-hair 25(OH)D_3_ concentrations. For example, although an 8-week interval between substance ingestion and detection is normally recommended in drug testing that targets hair samples (e.g.,^28^), it has been demonstrated that some drugs can be measured in beard hair between 1 to 14 days after the time of injection (e.g., three days in beard for cotinine saliva injection)^32,33^. SIA have also contributed to our understanding of the time-lag between the ingestion of food or water, and the detection of those in growing hair. In mammalian fur and human hair, hydrogen (*δ*^2^H) and oxygen (*δ*^18^O) isotope ratios are strongly correlated with that of source drinking water, albeit with contributions from water in food (both liquid and that formed from macronutrient catabolism) and atmospheric sources (see^34,35^, and Supplementary Information Table S1 for human hair and water *δ*^2^H and* δ*^18^O relationships). In an enriched ^2^H water drinking experiment on human beard hair, an increase in ^2^H first showed at 8 days and a plateau was reached at 20 days^35^. These results were similar to those of a study on the carbon isotopic composition of human beard hair which suggested that changes in the isotopic composition of diet can become apparent in 6–12 days, although had not reached equilibrium by 14 days^36^. In a study on human head hair, however, a migrant to Salt Lake City initially showed a change in their hydrogen and oxygen isotopic values in less than the 4 weeks’ sampling resolution but only approached a plateau at ~ 6 weeks^37^, suggesting that the isotopic ‘time-lag’ in head hair may be greater than for beard hair in humans. This was also the case in a 28-day controlled dietary study of four individuals, where the analysis of carbon and nitrogen isotope ratios in groups of sequentially-sampled head hair strands donated 5.5 months after the study reflected the dietary switch in all individuals, but where the isotopic signal of the dietary change was ‘smeared’ across multiple months of hair growth^26^: 2453). The authors attribute this extended time-lag not only to the anticipated turnover in the isotopic composition of proteins in the body available for hair growth, but also to the sampling strategy and to the fact that a cut lock of hair will incorporate hairs in different phases of growth^26^. Given uncertainties regarding the incorporation of 25(OH)D_3_ into hair, and the different time-lags evident from metabolite and stable isotope studies explored above, the period represented between the input levels and sampling of hair at the scalp could be anything between a few days and more than a month. However, regardless of the potential unknowns surrounding time-lag (i.e. between circulating serum vitamin D levels and vitamin D in new hair growth), the measurement of 25(OH)D_3_ taken at intervals throughout individual hair strands (or groups of strands/locks) has the potential to generate time-series insights into vitamin D status throughout the total growth period represented in the hair length and should be further investigated.

Using an optimised analytical methodology to measure 25(OH)D_3_ in hair intended to reduce sample size, our exploratory study aims to generate time-series vitamin D data for multiple modern participants from the same city (Aberdeen, Scotland, UK) through the sequential sampling of donated locks of hair (i.e. groups of individual strands) which had been cut from close to the scalp, aligned by cut end, and then segmented. In the first study of its type, we also undertake the same analysis on an archaeological individual from Aberdeen dating to the early modern period (16th/17th century AD; see Supplementary Information). A further objective of this study was to compare the intra-hair 25(OH)D_3_ data from the modern study participants to self-reported data accumulated via questionnaire during sample collection. For the longest locks of modern hair (*n* = 4), oxygen and hydrogen isotope data were also generated from cut serial-segments along with vitamin D data. This was done to determine whether this combined approach might highlight any potential seasonal relationship in *δ*^18^O or *δ*^2^H, and 25(OH)D_3_, due to the relationship between drinking water stable isotope chemistry and that of body tissues, and the fact that intra-annual variations in water isotopes should reflect local climatic conditions, including seasonal temperature (see^38,39^, and the Supplementary Information). For the archaeological sample, vitamin D data were also paired with the results of multi-isotope analysis (oxygen, hydrogen, carbon, nitrogen, sulphur) down the length of the lock of hair to explore any potential relationships between 25(OH)D_3_ concentrations, season and diet (i.e., trophic level, the consumption of marine fish).

## Results

25(OH)D_3_ data were obtained from 14 of the 16 participants, with two individuals having concentrations below the detectable limit in all hair segments. All 25(OH)D_3_ concentration data, along with corresponding stable isotope data, can be found in Supplementary Table S2, and similar data for the archaeological sample can be found in Supplementary Table S3. Table 1 summarises the baseline characteristics of the participants in this pilot study. While the participants with no detectable 25(OH)D_3_ data are discussed briefly below, these data points (along with some individual measurements below the detection limit for other participants) are excluded from the statistical analysis.

**Table 1:** Baseline characteristics of the participants (total population) of the individuals sampled as part of this study (*n* = 16) as well as average 25(OH)D_3_ concentration data (*n* = 14), and *δ*^18^O and *δ*^2^H (*n* = 4) data.

	Mean ± 1 SD	Range
Sampling month and year: Feb 2022 / March 2022 / April 2022 / May 2022 / Sept 2023	10 / 2 / 1 / 2 / 1	
Female / male (*n*)	11 / 5	
Age (years)	39.3 ± 12.6	18 – 58
BMI	23.5 ± 3.1	19.1 – 29.0
Caucasian / non-Caucasian (*n*)	15 / 1	
Estimated sun exposure (h / day)	1.4 ± 0.6	0.1 – 3 h
Vitamin D supplements yes / no (*n*)	9 / 7	
Oily fish consumption: weekly / 2–4 times a month / once a month / hardly or never (*n*)	2 / 8 / 4 / 2	
Hair colour: blond / brown / red / black (*n*)	3 / 10 / 2 / 1	
Total length of hair lock sampled: < 5 / 5–15 / 15–25 / 25–35 / 35–45 / 45–55 / 55–65 (cm; *n*)	2 / 4 / 3 / 2 / 0 / 4 / 1	
Mean segments analysed per person	13 ± 8	4 – 28
25(OH)D3 (pg/mg hair)	5710.3 ± 11,682.4	57.9 – 65,852.5
*δ*^18^O (‰)	11.2 ± 1.0	9.4 – 13.2
*δ*^2^H (‰)	-81.9 ± 3.4	-89 – -77

Data are reported as means, ± 1 standard deviation (SD), plus ranges, or number of individuals.

### Participant dataset—demographic trends

**Fig. 1:**
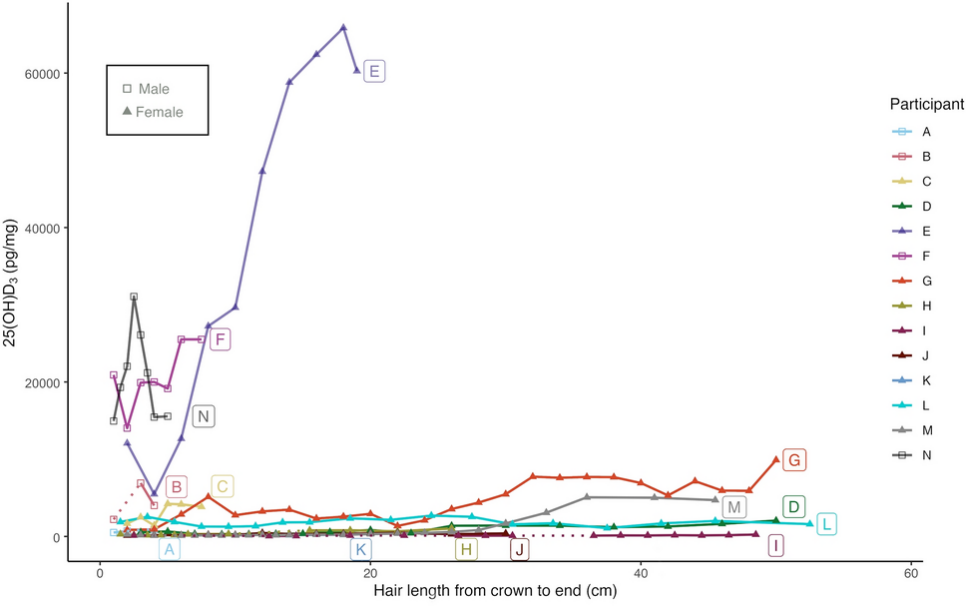
Intra-hair variation in 25(OH)D_3_ concentrations (pg/mg) from crown to end in cm, for participants (*n* = 14). Dotted lines have been added when 25(OH)D_3_ concentrations were below the detection limit.

Participants E (female), F (male), and N (male) had the highest overall concentrations of 25(OH)D_3_, with intra-hair values ranging from 5480.0 to 65,852.5 pg/mg, and with a median of 38,442.0 (IQR 43,588) pg/mg (participant E); 19,999.3 (IQR 3685) pg/mg (participant F); and 20,240.2 (IQR 7515) pg/mg (participant N), demonstrating variability in 25(OH)D_3_ levels down the total length of the sampled hair (Fig. 1). Median 25(OH)D_3_ concentrations for each of these three individuals were > 15,000 pg/mg higher than those of all other individuals in the study whose median concentrations did not exceed 4500 pg/mg but ranged from 57.9 to 9902.0 pg/mg. At the time of sampling, individuals E and F reported taking vitamin D supplements (although the strength/type of supplement was not recorded in the participant questionnaire), and E and F reported sharing the same household. Participant N reported that they did not take supplements of any kind. All three individuals reported major recent weight loss of 20–30 kg over the two years prior to sampling in the case of E and F, and 16 kg in the 3 months prior to sampling in the case of N. For the other participants, all 25(OH)D_3_ concentrations were below 10,000 pg/mg throughout the total length of the hair sampled, and two individuals, O (male) and P (female) (see Supplementary Table S2), had concentrations that fell below the level of detection for all hair segments.

**Fig. 2:**
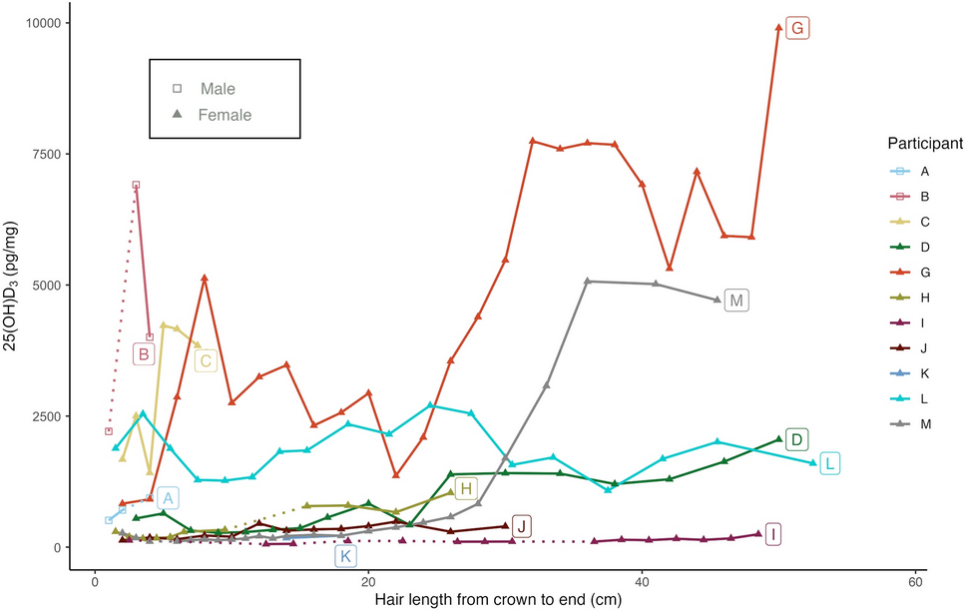
Intra-hair variation of 25(OH)D_3_ concentration (pg/mg) from crown to end in cm for individuals with intra-hair values of less than 10,000 pg/mg (*n* = 11). Dotted lines have been added when 25(OH)D_3_ concentrations were below the detection limit.

In order to better compare those individuals with lower average 25(OH)D_3_ concentrations, the three individuals with the highest values (E, F, and N) are excluded in Fig. 2 and from the statistical analyses which comprised of linear mixed-effects models. Due to the inability to assign any numerical values (including zero), the two individuals who had no detectable vitamin D were also excluded, along with those segments from other individuals that fell below the detection limit. None of the linear mixed-effects models had an Akaike Information Criterion (AIC) above the AIC of the null model, meaning that the tested variables had no effect on 25(OH)D_3_ concentrations (see Supplementary Information, Table S4). Comparison of 25(OH)D_3_ concentrations across individuals did not indicate any trends with age or sex; BMI category (with analysis excluding one individual who self-reported that they were pregnant); whether or not participants took vitamin D supplements; average time spent out of doors daily; or oily fish consumption (linear mixed-effect models, Table S4). However, although the number of segments analysed is relatively large, the number of individuals included in the study is small, and as such statistical analysis should only be considered exploratory and indicative-only. It should be noted that the two participants with no detectable vitamin D shared few baseline characteristics (see Table 1) other than being Caucasian and having brown hair (like the majority of study participants), and having reported spending 1 h/day outside, which is within the range of times reported by other individuals. Another individual (K) who had vitamin D levels above the detection limit in only 2 of 7 hair segments was the only non-Caucasian individual recruited for the study. However, the lack of ethnic diversity in the cohort; the self-reported nature of all the baseline data; and the lack of specificity in the questionnaire for some questions (e.g., time of day typically spent outdoors, strength of supplements if taken, etc.) represent significant limitations of this study.

### Participant dataset—intra-hair variability

A number of individuals demonstrated variability throughout the period of hair growth. This included all those three individuals with the highest values, two of whom display steeply declining values towards the period in which the hair was sampled in the case of E and F, and—in the case of N—steeply increasing and then decreasing values over approximately 5–6 cm of hair growth. In some individuals (e.g., G, L) possible seasonal trends were apparent, with peaks approximately 10–14 cm apart down cut locks of hair (a length of hair representing approximately 1 year of growth). Participants B and C showed variation over a shorter period of approximately 4–8 months, with inter-annual insights limited by the length of the hair available to sample. Other participants (e.g. M) demonstrated variability, but only in one part of the hair, with the only clear pattern being a drop in 25(OH)D_3_ from ~ 5000 pg/mg to around 200–100 pg/mg in hair approximately 30 cm from the scalp (i.e. ~ 2.5 years before sampling), a period corresponding to the outbreak of the covid-19 pandemic and the first lockdown in Scotland. Participants I, L and K demonstrated little intra-hair variation, although it should be reiterated that only two sub-samples of participant K’s hair yielded detectable levels of 25(OH)D_3_ (see above).

### Participants dataset—trends with stable oxygen and hydrogen isotope data

**Fig. 3:**
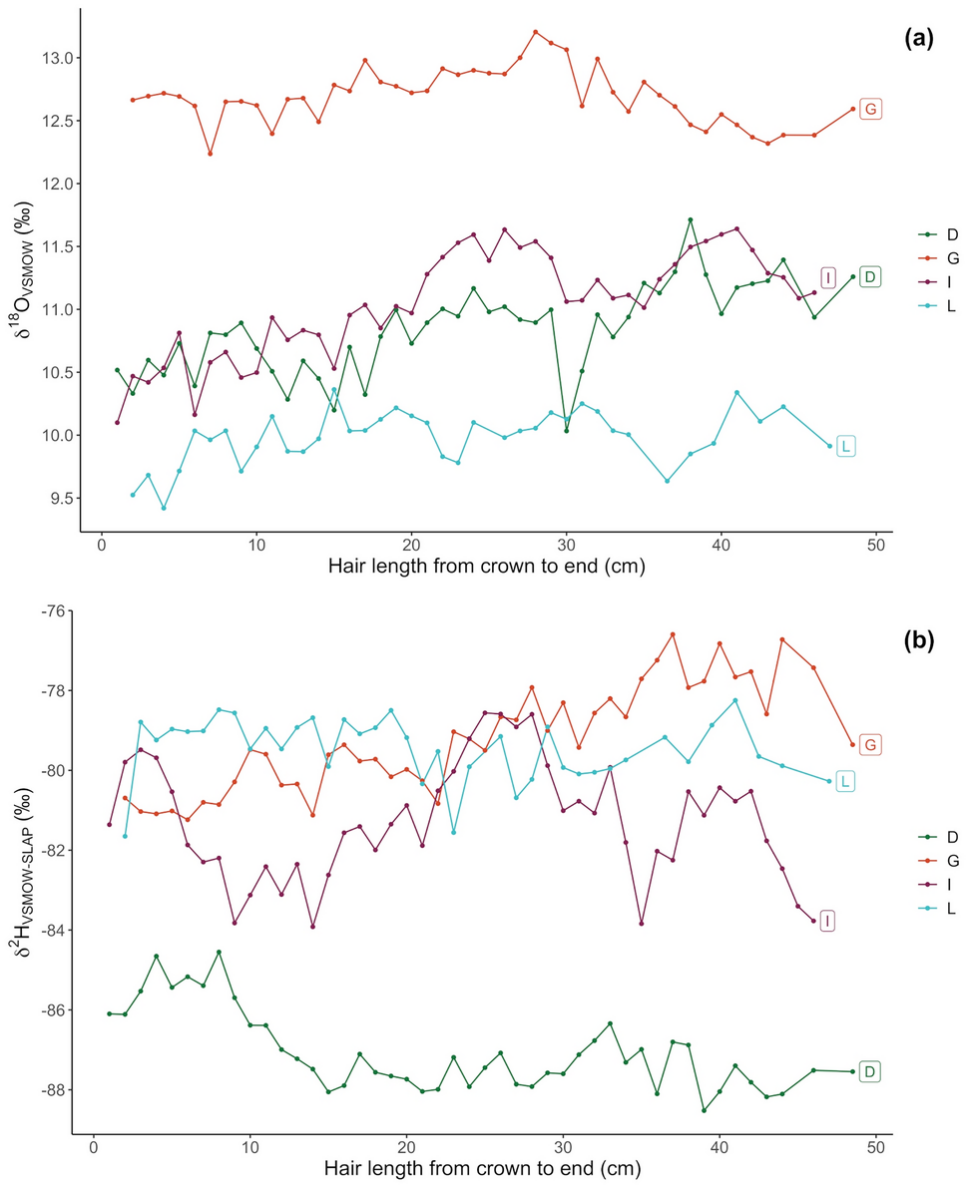
Intra-hair oxygen (**a**) and hydrogen (**b**) isotope measurements from crown to end in cm for four modern participants D, G, I and L.

Oxygen (*δ*^18^O) isotope values for four of the individuals (D, G, I, L) ranged from 9.4 to 13.2 ‰, and hydrogen (*δ*^2^H) isotope values ranged from -89 to -77 ‰ (see Supplementary Information Table S2 and Fig. 3). Inter-individual pooled means (i.e., the average of the mean of each of the four individuals) were 11.1 ± 1.1 ‰ and –81.8 ± 3.7 ‰ for *δ*^18^O and *δ*^2^H respectively (*δ*^2^H = -54.2 ± 4.8 ‰ when normalized to United States Geological Survey [USGS] human hair standard materials, USGS42-USGS43, see Supplementary Material). Averaged local groundwaters and freshwaters in this part of north-east Scotland have been estimated to be approximately -8 and -6 for *δ*^18^O and -55 to -40 for *δ*^2^H^40,41^. *δ*^2^H values in Aberdeen human hair are reasonably consistent with some but not all published hair-water relationships and with the eastern USA observations (see Supplementary Material for details): mean values are within 9 ‰ of the result calculated (using USGS42-USGS43 normalized data and water δ2H = -40 ‰,^42^) or within 7 ‰ of measured (compared to Massachusetts USA, water δ^2^H = -43 ‰,^43^). An offset is expected between *δ*^18^O drinking water and *δ*^18^O hair values. Based on local environmental *δ*^18^O values^40,41^, and based on published regression relationships between tap waters and human hair^37^, average predicted hair* δ*^18^O values for those living in and around Aberdeen should be between 12 and 13 ‰, estimates which are close to the calculated pooled mean and standard deviation. Hair samples from Cambridge and Brookline, Massachusetts and College Park, Maryland in the eastern USA have group mean *δ*^18^O values from 10.3 to 11.1 ‰, with tap water values of -6.0 to -6.9 ‰^43^, which agree with the hair and water values of the Aberdeen samples.

**Fig. 4:**
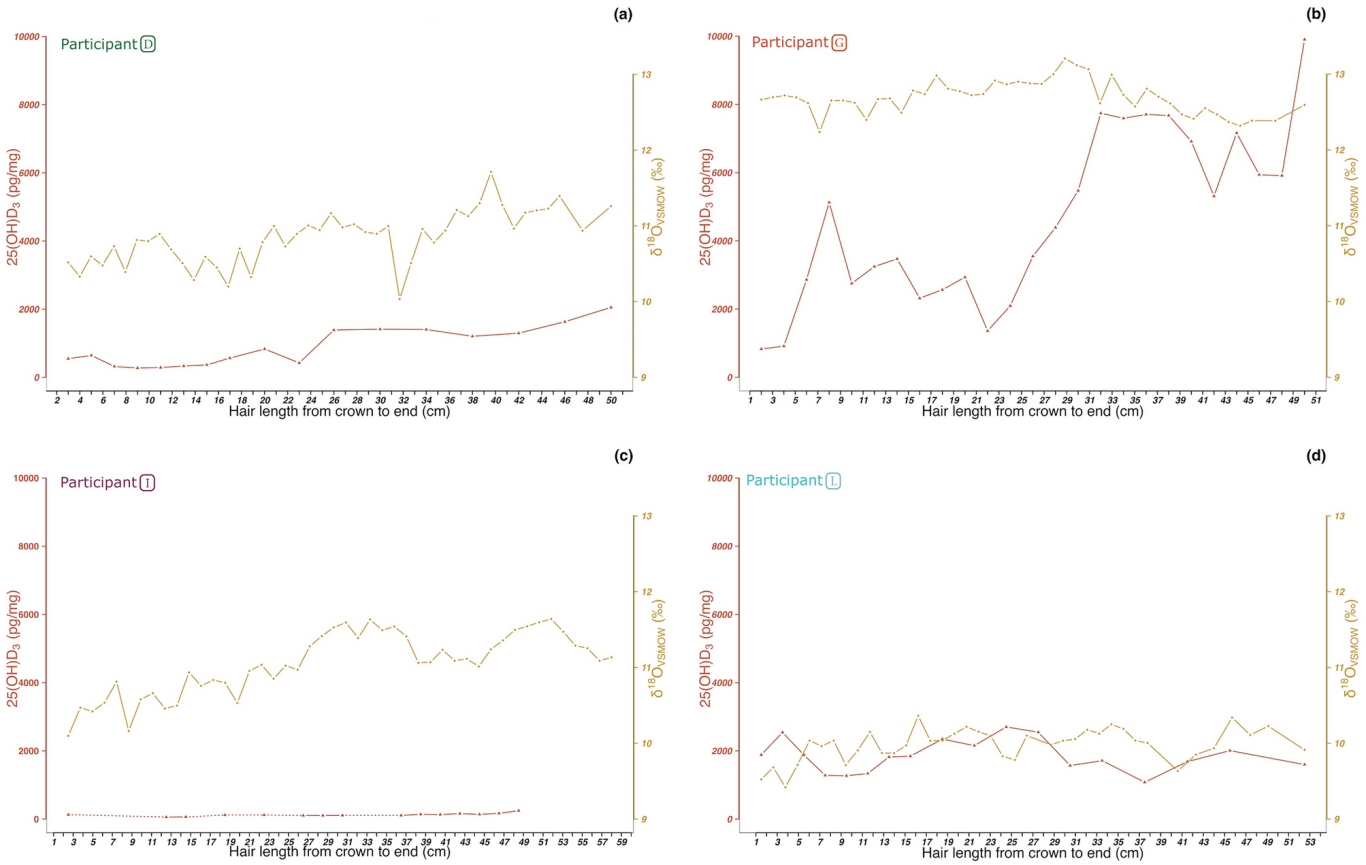
Intra-hair variations of 25(OH)D_3_ concentration (pg/mg) and oxygen isotope ratios from crown to end in cm of four participants. (**a**) Participant D; (**b**)  participant G; (**c**)   participant I; (**d**)   participant L.

All four individuals displayed some degree of intra-hair variability across both their oxygen and hydrogen isotope ratios (see Fig. 3a, b). Although seasonal signals were attenuated compared to modelled local seasonal variation in precipitation^44^, in the case of *δ*^18^O, subtle seasonal variations could be observed across all individuals (Fig. 3a), with the clearest seasonal patterns in participants D and L (Fig. 3a). In all participants, and particularly in participants I, L, and D, the oxygen isotope measurements within ~ 4 cm of the scalp were on a downward trend (through time), corresponding to the lower values anticipated for winter or early spring. As these hair samples were all taken between February and May 2022, this could be consistent with the anticipated time-lag between dietary (water) inputs and tissue ‘outputs’^37^. Comparison between intra-hair variability in *δ*^18^O and vitamin D concentration in individuals G, L and D provided some tentative evidence for a co-varying relationship between season (as indicated by *δ*^18^O) and vitamin D concentration in hair (Fig. 4 a, b, c and d), with those vitamin D levels within ~ 4 cm of the scalp also on a downward trend. Statistical description of this relationship is challenging, due to the unknowns surrounding the (a)synchronicity of vitamin D uptake in hair compared to protein synthesis. Additionally, vitamin D and stable isotope analyses were conducted on the same segments (as opposed to adjacent segments) in only two of the four individuals (participants D and G). When vitamin D and *δ*^18^O are compared for these two individuals, Spearman’s rank correlation does suggest a statistically significant positive correlation between these two variables (coefficient S = 3474; *p*-value = 3.32e^-05^; ρ = 0.62). However, when analysed separately, the positive correlation between vitamin D and *δ*^18^O is no longer significant (Individual D [Coefficient S = 489.31; p-value = 0.2928; ρ = 0.28]; Individual G [Coefficient S = 2385; *p*-value = 0.1139; ρ = -0.35). While this may be a function of reduced sample size, samples from a greater number of individuals would be required to better characterise the relationship between vitamin D and *δ*^18^O in modern hair.

Hydrogen isotope profiles were even more complex (see Fig. 3b), and while one individual had a clear sinusoidal pattern (participant I), other intra-hair trends were more unpredictable (i.e. gradually increasing values throughout hair growth in individual D, and gradually decreasing values in G). In one individual, intra-hair variability in *δ*^2^H barely exceeded analytical precision (individual L). Statistical analysis revealed the relationship between vitamin D and *δ*^2^H in both individuals D and G to be significant, although the directionality of the relationship was inverted. Participant D had a positive correlation (Spearman’s Rank Correlation Coefficient S = 1078; *p*-value = 0.01724; ρ = -0.59), and for participant G vitamin D and δ^2^H were negatively correlated (Coefficient S = 377.66; *p*-value = 1.407e^-05^; ρ = 0.79).

### Archaeological sample—multi-isotope and vitamin D data

**Fig. 5:**
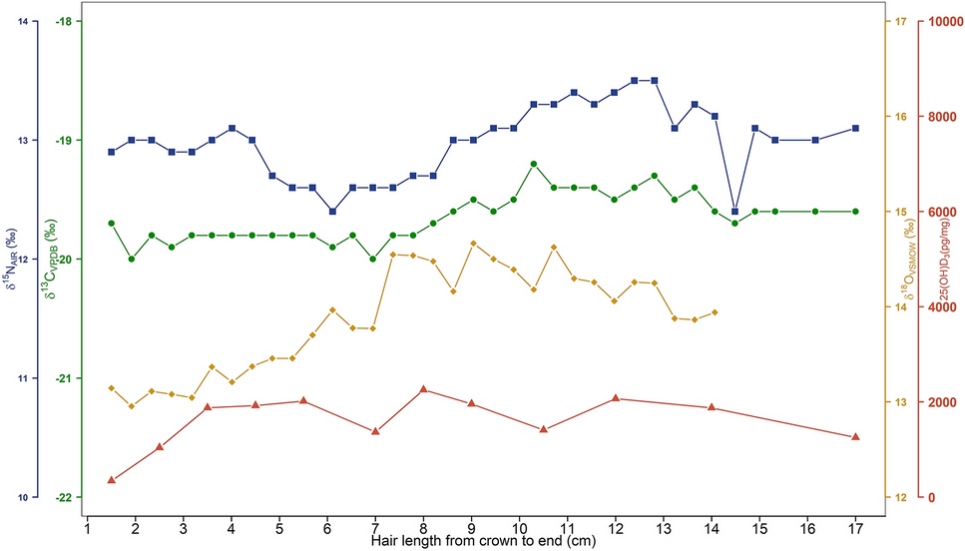
Multi-isotopic and 25(OH)D_3_ intra-hair variations from crown to end in cm for the archaeological sample from St Nicholas Kirk (Aberdeen, Scotland).

The archaeological sample (SN-243) from St Nicholas Kirk, Aberdeen, displayed 25(OH)D_3_ concentrations that ranged from 342.6 to 2254.6 pg/mg (Fig. 5) across the ~ 17 cm lock, corresponding to around a year and a half of growth. Vitamin D concentrations are within the range of the majority of modern Aberdeen hair samples (see Fig. 2, and the Supplementary Material), while *δ*^18^O (and *δ*^2^H) isotope ratios were slightly elevated compared to the modern population and demonstrated a greater intra-hair variability. Oxygen isotope data from the archaeological sample, an adult male (see Supplementary Material), were most similar to modern participant G. Modern participant G reported living on a private water supply.

As shown in Fig. 5, the intra-hair *δ*^18^O isotope data from the St. Nicholas Kirk individual co-varied with 25(OH)D_3_ concentrations, with the lowest 25(OH)D_3_ concentrations coinciding with the lowest *δ*^18^O values. Statistical analysis suggests this relationship to be marginally significant (*p*-value = 0.05258; ρ = 0.5968; Spearman’s Rank correlation coefficient = 88.701), suggesting possible seasonal variability in 25(OH)D_3_. Peaks in both *δ*^13^C and *δ*^15^N isotope ratios in the segments from the middle portion of the total length of hair sampled (between approximately 14 cm and 8 cm from the scalp; see Fig. 5) may correspond with the increased consumption of marine or anadromous protein in the summer before death. Sulphur isotope ratios did not vary and were elevated throughout the hair shaft (between ~ 18 and 19 ‰, see Supplementary Information Table S3), consistent with values of those dwelling in coastal locations, due to the sea spray effect^45^.

## Discussion

In this study, 25(OH)D_3_ has been successfully extracted from human hair from 14 out of 16 modern participants and a single archaeological individual, and concentrations have been determined. Concentrations reported for the majority of samples included in this study overlap with, but generally exceed, ranges reported in previously-published studies (e.g., 11.9 to 911 pg/mg in^14^,9.31 to 1541 pg/mg in^13^,and 15 to 2000 pg/mg in^12^). Our elevated concentrations are likely the result of several factors, including the baseline characteristics of the individuals in our study (see Table 1) and our segmental sampling strategy which has perhaps served to capture more variation than ‘bulk’ sampling most commonly employed elsewhere. Most notably, however, here we utilise a modified extraction protocol which incorporates butylated hydroxytoluene (BHT). Due to the instability of vitamin D and its analogues, BHT or other antioxidant agents are commonly added during determinations on a variety of matrices as these inhibit lipid degradation and lipid oxidation^46^. The use of BHT during extraction from human hair has perhaps served to maximise yields through the preservation of vitamin D during extraction. This has, in turn, allowed for the analysis of smaller samples in relatively larger methanol extraction volumes, optimising extraction kinetics and preventing saturation. This represents an important methodological development ahead of the application of this technique to finite archaeological samples, and/or when studies wish to incorporate tandem approaches, such as stable isotope analysis. However, due to differences in extraction protocols, the comparison of the empirical data obtained in the small number of studies published on hair to date (including the current study) remains a challenge. Indeed, differences in analytical protocols remain one of a number of challenges in the emerging field of archaeometabolomics^16^. While we include spiked standards during extraction, which should enhance methodological comparability across studies, we would recommend that future studies on human hair include the preparation of a commercially available (homogenized) human hair standard (such as USGS42 or USGS43) to allow extraction efficiencies to be accounted for.

For the majority of individuals included in this study, the measurement of sequentially-sampled hair segments from modern participants from Aberdeen, Scotland, has revealed variations in 25(OH)D_3_ along the length of the sampled locks. This was also the case for a lock of hair sampled from an early modern (16^th^/17^th^ century AD) male buried in the city of Aberdeen. The time-series changes in 25(OH)D_3_ concentrations we determined are not consistent with the wash-out effect sometimes observed with other secosteroids (e.g., cortisol,^47^). Our findings thus support and extend the outcomes of other studies that have detected and quantified vitamin D in human hair^12–14^. The fluctuations in 25(OH)D_3_ concentrations observed across many of the samples in our study appear consistent with the bioaccumulation of 25(OH)D_3_ in hair in varying concentrations during tissue growth. The graded intra-hair increases and decreases in 25(OH)D_3_ we observed in many of the participants are therefore perhaps the best evidence to date that measurements of 25(OH)D_3_ in human hair obtained via LC/MS correspond to *in vivo* deposition during hair growth. We also demonstrate the validity of the methodological approach in acquiring 25(OH)D_3_ from archaeological hair and highlight the potential for this method to extend investigations of vitamin D status in archaeological populations beyond the identification of skeletal manifestations of deficiency towards empirical estimates of vitamin D status that may eventually be possible through archaeometabolomics. It is also of note that vitamin D concentrations in the archaeological sample were similar to the majority of individuals from modern Aberdeen, despite (presumably) substantial lifestyle and dietary differences between the early modern period and the present day. This may suggest that local geographical factors related to UVB exposure (i.e. latitude) may be strong predictors of hair 25(OH)D_3_ concentrations, as has been determined in some studies on serum 25(OH)D_3_ (e.g.,^48^).

However, despite all modern individuals participating in this study having lived in the same area (North-East Scotland) for at least 2.5 years prior to hair sampling, there was a high coefficient of variation in 25(OH)D_3_ between some participants, and even amongst those reported to be living in the same household. When examining interpersonal differences, weight loss appears to play a major role in the 25(OH)D_3_ concentration of hair. Three of the modern participants demonstrated average values that were higher than other participants by a factor of ten or more in most cases. Participants E and F, a male and female who shared the same household, reported weight loss of > 20 kg during 2020 and 2021 (periods that would be represented in the growth of the cut hairs from each individual). Participant N also reported a rapid weight loss of 16 kg in the months immediately preceding sampling in 2023. It has been previously established that vitamin D bioaccumulates in adipose tissue, leading to lower circulating vitamin D levels in obese individuals, and also that vitamin D levels increase following adherence to a very low-calorie ketogenic diet due to the release of stored vitamin D^49^. Data observed in patients with obesity who had recently undergone bariatric surgery have also demonstrated increased serum 25(OH)D_3_ soon after procedures^50,51^. We suggest that the elevated but decreasing values observed in the hair of individuals E and F in our study are due to sustained long-term fat loss and that the sudden increase, and then decreasing values seen in participant N may be consistent with the sudden, acute weight loss followed by weight stabilisation reported by that individual.

Beyond recent (major) weight loss, no other trends were determined between hair 25(OH)D_3_ concentrations and the baseline characteristics captured in our questionnaire including oily fish consumption, hours spent out of doors, age and sex, or whether or not individuals reported taking supplements. This is surprising considering the strong relationships that have been noted between blood 25(OH)D_3_ and oily fish consumption, for example, in some studies (e.g.,^4,52^). However, our analysis is limited by the small number of participants and the related lack of statistical power. Furthermore, the retrospective self-reporting used in our study represents another limitation. For example, participants were asked to report their weight but were not provided with scales. There was also variation in what was reported, with some choosing to add significant additional information (e.g., on recent weight loss patterns). Furthermore, some of the questions posed have proven insufficiently nuanced: for example, although participants were asked ‘Do you currently take daily vitamin D supplements, or multivitamins containing vitamin D?’, they were not asked about the strength of any supplements taken. In future studies involving modern participants, a more comprehensive questionnaire that tracks daily food consumption in the weeks (or months) prior to hair sampling, and/or a controlled dietary intake (i.e. where meals or supplements are provided), for example, could help to better test for any correlation between 25(OH)D_3_ in hair and supplementation, oily fish consumption, etc. In the case of fish consumption, isotopes of carbon and nitrogen could also be employed to independently test for habitual fish consumption in modern participants. Indeed, a correlation between serum 25(OH)D_3_ levels and *δ*^15^N values in red blood cells due to the consumption of marine resources has already been documented^53^, and – as with our archaeological individual – this approach could be employed to explore the relationship between fish consumption and vitamin D levels in future studies of 25(OH)D_3_ in modern human hair.

However, a limitation to any future studies is that the empirical relationship between serum/plasma 25(OH)D_3_ concentrations and that of growing hair remains unknown, making the interference of vitamin D status (i.e. deficiency) in the individual challenging. While one study has reported a lack of correlation between serum and hair 25(OH)D_3_ levels^12^, this study was not sufficiently powered and the experimental design did not account for the expected time-lag between circulating blood 25(OH)D_3_ concentration and that of hair. Although human head hair grows continuously and a growth rate of ~ 1 cm per month is widely accepted^28,29^, here we undertook *δ*^18^O and *δ*^2^H analysis to better understand the periodicity of hair growth and to attempt to relate intra-hair variation in vitamin D to seasonality. *δ*^18^O and *δ*^2^H vary seasonally in precipitation at mid-high latitudes, with elevated values anticipated in summer, and lower values anticipated in winter. Although tap water stable isotope ratios have lower intra-annual variability compared to local precipitation due to factors such as storage volumes and lack of seasonal recharge, serially-sampled human hair from urban centres have previously been demonstrated to reflect (buffered) seasonal variability in water stable light isotope composition^43,54,55^. The oxygen isotope data presented here from four of the participants are consistent with these findings, and are also consistent with the timing of sampling, taking into account a delay of a month or more from isotopic input to tissue synthesis. Possible seasonal relationships between oxygen isotopes and vitamin D were identified in some individuals, with vitamin D and *δ*^18^O being positively correlated (significantly so, when considering paired vitamin D and *δ*^18^O data all co-analysed modern samples collectively, as well as for the archaeological individual). This appears to lend tentative support to the notion that hair, like blood serum^5^, may reflect seasonal changes in vitamin D levels related to UVB exposure during the summer months. These potentially seasonal relationships also highlight that there may indeed be a considerable offset between dietary (or dermal-synthesised) ‘inputs’ and vitamin D concentration ‘outputs’ in hair, and a long-term paired-tissue (serum-hair) study would be required to better understand and characterise this relationship.

In comparison to oxygen isotope ratios, intra-hair trends in hydrogen isotope ratios in this study were less clear, with only one individual showing a clear sinusoidal intra-hair trend. Statistically significant relationships between 25(OH)D_3_, and *δ*^2^H were noted in two individuals, however, correlations were positive in one case, and negative in the other. This highlights that hydrogen (and oxygen) isotopes in hair reflect multiple inputs to tissue chemistry, including drinking water, but also food water and (in hydrogen to a greater extent than oxygen) structurally-bound food-derived H or O (i.e. from diverse, store-bought foods). Observational studies in humans have shown less of a distinction in food *versus* water contributions between hydrogen and oxygen. Based on the slope of the regression or two-point comparison between the isotopic composition of hair and water most estimates (excluding^56^) of the amount of H from drinking water reflected in hair are 27–49% (see Supplementary Information, and^35,42,57,58^) and for oxygen are 27–40%^37,58,59^. However, a recent model has estimated that 60% of hydrogen in animal keratin is routed from diet, compared to 19% from oxygen^60^. Further studies are required to better understand these relationships, and how both oxygen and hydrogen isotope ratios in hair can be used to seasonally ‘anchor’ other types of data, including that generated by metabolomics.

In the case of the archaeological individual, there was, however, a pronounced variability in intra-hair oxygen isotope ratios and a strong apparent relationship between season and vitamin D. This is likely consistent with the post-medieval water supply of the city of Aberdeen being more closely related to seasonal variation in precipitation than today’s supply. Previous studies have demonstrated that oxygen and hydrogen isotope ratios of hair from archaeological samples can be used to estimate the season of death^61^. In the archaeological individual from St. Nicholas Kirk, the oxygen isotope data closest to the scalp relate to the coolest months, which (given the time-lag discussed above) makes it likely that the individual died in spring, coinciding with the seasonality of a number of common pathogens^62,63^ that could have proved fatal in the early modern period. This first archaeological application serves to highlight the potential for this technique to be developed to explore past health, and the relationships between certain types of foods and vitamin D status in the past, particularly amongst high latitude populations where exposure to sunlight is low and dietary intake from sources such as marine foods may be crucial. However, prior to the further development of this methodology as either an archaeo-investigative tool or a non-invasive test to determine vitamin D levels in modern cases, the relationship between serum 25(OH)D_3_ concentrations and that of hair must be ascertained. Further exploration, including expanded sample sizes and planning a long-term paired-tissued (serum-hair) study, will be crucial to unravelling the intricate mechanisms underlying vitamin D integration into hair and its broader implications.

## Methods

### Sampling protocol

This pilot study involved both modern human participants and a single archaeological sample, both originating from Aberdeen in the United Kingdom. Hair sampling was conducted at the Rowett Institute, Aberdeen, United Kingdom, in 2022 (February to May) and 2023 (September), with participants recruited from students and staff at the University of Aberdeen using posters. The study was carried out in accordance with the ethical principles of the Declaration of Helsinki, Good Clinical Practice, and complied with the requirements of the Data Protection Act 2018 (applying to the UK’s GDPR standards). Ethical approval was obtained from the Rowett Ethics Panel prior to commencing the participant recruitment, sampling, and analysis. All participants provided written informed consent before starting the study. Participants of any gender aged 18–75 years, with hair length coming from the posterior vertex region of the scalp being at least 2.5 cm long, and who had lived in north-east Scotland for at least the past 2.5 years, were recruited. Exclusion criteria were having hair that was dyed, permed, bleached or otherwise chemically treated. Each participant completed a short questionnaire regarding their age, body weight, height, hair colour, oily fish consumption, average hours spent outside every week, and vitamin D supplementation (see Supplementary Information Appendix for a blank example of the short questionnaire). All information was self-reported, and a number of individuals volunteered more detailed information in the blank spaces. For example, several individuals reported recent weight loss, with dates; three individuals reported cohabitating with one another; one individual self-reported pregnancy; and another individual specified the date they had moved to Aberdeen 2.5 years before the sampling. As the back of the head has the most uniform hair growth^47^ groups of strands were cut in this area as close as possible to the scalp. Hair length varied between 4 and 58 cm, and samples consisted of approximately 15–30 strands per individual taken as a single ‘lock’ of hair. Groups of strands were then separated into two aliquots, one for vitamin D extraction, and (if a sufficient amount remained) one for isotope ratio analysis. This process was repeated for the archaeological sample, which had been obtained close to the crania of a single male inhumation from the Kirk of St. Nicholas, Aberdeen. Prior to further preparations, the archaeological sample was first ultrasonicated in deionized water (15 min; × 2) to remove adhering soils or other particulate from the burial environment, and left to dry.

### Vitamin D extraction and analysis

Modern hair strands set aside for vitamin D analysis were aligned by cut end; sliced into 0.5 cm segments (variably referred to as ‘serial’, ‘segmental’ or ‘sequential’ sampling in the literature) using a scalpel, ruler, and non-slip polyvinyl cutting board; and weighed into microcentrifuge tubes. Samples of < 1 mg were combined prior to extraction, with mass largely dependent on the number of each hair strand in the lock and the thickness of the strands. Due to the smaller quantity of material available, the archaeological sample was cut into sections of between 1 and 3 cm in length, and (combined) sample weights ranged from 1 to 24 mg for the segments (see Supplementary Information Table S3). Archaeological and modern hair samples were prepared for vitamin D analysis using a modified method based on a previous study^14^ in batches of 20–40 samples. Each sample (or combined sample) was placed in a microcentrifuge tube and washed twice with 2 mL of isopropanol at room temperature. Following the wash, 1.4 mL of methanol containing 1 mg/mL of butylated hydroxytoluene (BHT) was added, along with 10 µL of an equal-parts mixture of 25(OH)D_3_ and 1,25(OH)D_3_ (stable isotope labelled) internal standards. Samples were subsequently placed on an agitator overnight at room temperature. The extracts were then transferred to new microcentrifuge tubes and evaporated under a nitrogen stream at 50 °C until dry. After this, the samples were reconstituted into 50 µL ethanol for LC–MS/MS analysis at the Rowett Institute (University of Aberdeen, Scotland). The LC–MS/MS analyses were performed in duplicate on an Agilent 1290 Infinity UHPLC connected to an Agilent 6490 triple-quadrupole mass spectrometer. For each measurement, 4µL of the prepared samples were injected onto an Agilent Poroshell column (3.0 × 50 mm, 2.7 µM) and separated in a 6-min isocratic method with a mobile phase consisting of 5 mM ammonium acetate + 0.1% acetic acid and Methanol (15:85). The 500µL/min flow was admitted directly into the Atmospheric Pressure Chemical Ionisation (APCI) source, in positive ion mode, and 25(OH)D_3_ & 1,25(OH)D_3_ were quantified by Multiple Reaction Monitoring (MRM). The quantifier transitions for 25(OH)D_3_ and 1,25(OH)D_3_ were, 383.2–365.2, and 399.2–381.1 respectively, and qualifier transitions were added to confirm identification. Calibration standards, ranging from 2 ng/µL to 10 pg/µL, with an internal standard concentration of 400 pg/µL, were prepared fresh and run prior to sample analysis, and the calibration curves generated were used to calculate the concentration of the 25(OH)D_3_ and 1,25(OH)D_3_ (pg/μL ) in the samples. Assay quality was monitored using in-house standards (coefficient of variation for 25(OH)D_3_ = 2.3%, 1 s.d. = 1.5, 1,25(OH)D_3_ = 2.1%, 1 s.d. = 1.5); and through the duplicate analysis of the samples (coefficient of variation, *n* = 90 = 3.5%) during the course of the experiment.

Of the samples analysed via mass spectrometry (*n*[individuals] = 16), 25(OH)D_3_ concentrations above the detectable analytical level were determined for 14 individuals. 1,25(OH)D_3_ was not detectable in the majority of samples, and is therefore not further discussed here.

The 25(OH)D_3_ content (pg/mg) of each hair sample was calculated using the following equation:

$${\text{C}}_{{\text{h}}} \left( {{\text{pg}}/{\text{mg}}} \right) \, = {\text{ C}}_{{{\text{ms}}}} \left( {{\text{pg}}/\mu {\text{L}}} \right) \, \times {\text{ V1 }}\left( {\mu {\text{L}}} \right) \, /{\text{ M}}_{{\text{h}}} \left( {{\text{mg}}} \right)$$

where C_h_ is the calculated amount of 25(OH)D_3_ in hair (in pg/mg); C_ms_ is the calculated amount of 25(OH)D_3_ obtained by LC–MS/MS (mean of duplicate analysis, in pg/μL); V1 is the reconstituted volume for LC–MS/MS analysis (= 50 µL); and M_h_ is the mass of the hair sample (in mg). Please note, as our extraction and evaporation volumes were the same, we do not include these in our equation.

From the 14 (modern) individuals from which 25(OH)D_3_ data had been collected, samples from four individuals were then also analysed for stable isotope composition.

### Stable isotope analysis

Modern and archaeological hairs were prepared for isotope analysis using standard protocols to degrease and clean samples based on O’Connell and Hedges^64^ and O’Connell et al.^65^. Individual locks were ultrasonicated in 2:1 chloroform/methanol solution (30 min; 2x) and then 1:2 chloroform/methanol solution (30 min; 2x) to remove lipids. The samples were then rinsed by ultrasonication in Milli-Q water (18 MΩ cm^−1^) (30 min; 2x). The cleaned hair from the modern participants was then cut into segments of various lengths depending on the remaining sample weight (see Supplementary Information Table S2 for lengths) and placed in individual microcentrifuge tubes. The archaeological hair sample, which was 17 cm in length, was cut into 0.5 and 1 cm sections ahead of isotope analysis. The segments were then freeze-dried before being analysed via mass spectrometry at the Department of Geosciences, Boise State University (*δ*^18^O and *δ*^2^H) and (in the case of the archaeological sample) at the Scottish Universities Research Centre (SUERC) for *δ*^13^C, *δ*^15^N, and *δ*^34^S (see the Supplementary Information for details of analytical protocols, standards, etc.). A glassy carbon-packed reactor was used for hydrogen and oxygen isotope ratio measurements, and *δ*^2^H and *δ*^18^O values are reported normalized to VSMOW on the VSMOW-SLAP scale, using aliquots of these water standards in sealed silver capsules directly.

### Statistical analysis

To investigate whether 25(OH)D_3_ concentrations (mass corrected, in pg/mg) varied with the different characteristics recorded by the participants, linear mixed-effect models were applied to account for the fact that we had multiple measurements from the same individuals. The random variable was defined as the participant ID, and since 25(OH)D_3_ concentrations were not normally distributed (Shapiro–Wilk normality test, *p* < 0.01), a log transformation was applied. Different models were created, and the Akaike Information Criterion (AIC) was used for model selection (see Supplementary Information Table S4). *Model 1* included all physical characteristics (sex, age, and BMI), while *Model 2* included all lifestyle characteristics (vitamin D tablet consumption, sun exposure, and oily fish consumption). *Model 3* combined all variables from models 1 and 2. The null model (intercept only) was compared to the other models, and only the model with an AIC lower than that of the intercept-only model were considered significant. All data analyses were implemented in R software (version 4.4.1) using the “lme4”^66^ package. For all the mixed models, we provided parameter estimates (β) with standard error (SE) and the 95% confidence interval (95%CI). All models met the assumptions of normality of the residuals and homogeneity of variance. Unless otherwise stated, the data is reported as median (IQR). To assess the relationship between 25(OH)D_3_ concentrations and stable oxygen and hydrogen isotopes in both the modern and archaeological individuals, Spearman’s Rank correlations were applied, while normality of the variables were checked using a Shapiro–Wilk test (*p*-value < 0.05). The correlations were considered significant when *p*-value ≤ 0.05.

## Supplementary Information

Supplementary Information.

Supplementary Tables.

## Data Availability

All vitamin D and stable isotope data generated in this study are publicly accessible in the associated Supplementary Information. All potentially identifying information collected during the study are not included.
