# Supplementary material for: Segmental analysis of human hair reveals intra-annual variation in 25(OH)D3 concentrations in modern and archaeological individuals
Source: Sci Rep. 2025 Jan 24;15:3078. doi: 10.1038/s41598-025-86097-6 (PMC11760916; doi:10.1038/s41598-025-86097-6)
Supplement: Supplementary file 1 — Supplementary Information. [file 41598_2025_86097_MOESM1_ESM.docx]

**Supplementary Information**

Britton, Czére, Gutierrez, *et al.* Segmental analysis of human hair reveals intra-annual variation in 25(OH)D_3_ concentrations in modern and archaeological individuals

N.B. Tables S2, S3 and S4 can be found in the related spreadsheet.

**1. Background and context: archaeological sample**

The church of St. Nicholas is positioned within the centre of New Aberdeen atop a small hill above the Denburn estuary, historically having occupied a dominant position in the Aberdonian landscape (Hunter 1972). Although the first written document referring to the kirk, a papal bull, dates to 1157 AD the church itself probably predates this charter; its origins have been suggested to date back to 1060 (Hunter 1972; Oram 2016). Rapid development along with Aberdeen as an urban centre directly influenced the growth of the church itself. By the 15^th^ century, St. Nicholas was one of the largest burgh churches in Scotland and the mid-16^th^ century became elevated as a collegiate church, gaining a degree of self-governance (Oram 2016).

Due to the redevelopment of the church in 2006, an excavation was carried out by the City Council Archaeological Unit. Multiple burial phases found here can be broadly grouped into pre-15^th^ century (Phase A) AD and post-15^th^ – 18^th^ century AD (Phase B) burials, although the majority of burials in Phase B likely date primarily 16^th^ and 17^th^ centuries (Britton et al. 2018a). These excavations produced an extensive skeletal assemblage providing the first opportunity to study diachronic population level information from the 12^th^ to the 18^th^ centuries AD in a Royal Burgh of North-East Scotland. Previous studies conducted on this assemblage address changes in breastfeeding practices (Britton et al. 2018a) and adult diet and mobility (Czére 2020; Kate Britton, unpublished data), along with previous and ongoing osteoarchaeological and palaeopathological analysis (Duffy et al. 2008; and Marc Oxenham and Rebecca Crozier, unpublished data). SN-243 dates to the post-medieval (Phase B) phase of the St Nicholas Kirk burial ground and has been the subject of prior and ongoing osteoarchaeological investigations. Based on standard osteological parameters, the individual was determined to be a male between 25 and 29, with an estimated stature of 177.56 ± 2.99 cm (Duffy et al. 2008). The results of the stable isotope analysis of (rib) bone collagen from this individual are similar to many individuals analysed from St. Nicholas Kirk Phase B, and are typical of regular consumption of marine fish as part of a diet based on terrestrial resources (*δ*^13^C = -19.4 ‰; *δ*^15^N = 13.5 ‰). Bone collagen sulphur isotope data are typical of many individuals analysed from the site (*δ*^34^S = 14.5 ‰) and of individuals living in coastal areas of north-east Scotland (Kate Britton, unpublished data). Hair strands also survived in the burial environment and were recorded still attached to the cranium. A section of hair was sampled in 2017 and kept at -20°C at the Archaeological Chemistry laboratory at the Department of Archaeology, University of Aberdeen, prior to analysis.

**2. Background: Oxygen and hydrogen isotope analysis of hair**

Oxygen and especially hydrogen isotopic analysis of keratins has been used for many years for provenance and migration studies, particularly hydrogen isotopes in bird feather or claw keratin (Hobson and Wassenaar 2008). The underlying principle is that geospatial variation in *δ*^2^H and *δ*^18^O in water (due to changes in temperature, altitude, distance from the oceans, etc., see Sharp 2017) is incorporated into tissues through the drinking water and local food consumed. Similar work on human hair has been developed partly for forensic and identification purposes (e.g., Bataille et al. 2022; Lehn et al. 2015) and to investigate the systematics of incorporation of isotope ratios from food and water into proteinaceous tissues. Several studies have determined the relationship between a location’s precipitation or drinking water (usually tap water) hydrogen and oxygen isotope values in human hair, as summarized in Table S1. The approach to data normalization is also given; this has varied as different reference materials were made available, including hair such as USGS 42 and USGS 43 and other keratin materials, and VSMOW and SLAP waters in sealed capsules which can be run alongside solid samples (also from the USGS, the United States Geological Survey).

Table S1. Human hair and water *δ*^2^H and *δ*^18^O relationships, and analytical methods used.

| **hair-water relationship^a^** | **normalization & measurement** | **note** | **reference** |
| --- | --- | --- | --- |
| *δ*^2^H_h_= 0.45 *δ*^2^H_w_ -45 | VSMOW with keratin materials KHS, CBS, USGS 43, USGS 42 | hair from Mexico, USA, Canada; includes re-normalized data from Ehleringer et al. 2009 | (Bataille et al. 2022) |
| *δ*^2^H_h_= 0.271 *δ*^2^H_w_ -79  *δ*^18^O_h_= 0.353 *δ*^18^O_w_ +15.2 | VSMOW using comparative reference materials (horse hair); glassy carbon reactor; non-exchangeable H | hair from across USA | (Ehleringer et al. 2008) |
| *δ*^2^H_h_= 0.42 *δ*^2^H_w_ -75  *δ*^18^O_h_= 0.40 *δ*^18^O_w_ +16.4 | VSMOW using comparative reference materials (horse hair); glassy carbon reactor; non-exchangeable H | hair from China, India, Mongolia, Pakistan | (Thompson et al. 2010) |
| *δ*^2^H_h_= 0.49 *δ*^2^H_w_ -35 | VSMOW using IAEA-CH-7 polyethylene and in-house materials; glassy carbon reactor; both total and non-exchangeable H given | hair from 11 locations, including USA, Eurasia, South Africa | (Fraser and Meier-Augenstein 2007) |
| *δ*^2^H_h_= 0.78 *δ*^2^H_w_ -49.5  *δ*^18^O_h_= 0.70 *δ*^18^O_w_ +19.2 | VSMOW using comparative reference materials (horse hair); glassy carbon reactor; non-exchangeable H | hair from mid-20^th^ century Indigenous populations | (Bowen et al. 2009) |
| 36% H from water  27% O from water | VSMOW using BWBII, CHS (Hoof Keratin Standard), CFS (Feather Keratin Standard); non-exchangeable H | hair from East Greenbush, New York, USA; and Fairbanks, Alaska, USA | (O'Brien and Wooller 2007) |
| 31% H from water | SMOW with NBS 30 biotite | hair from one person consuming ^2^H-enriched water | (Sharp et al. 2003) |
|  | VSMOW & SLAP directly; glassy carbon reactor; total H | hair from eastern USA | (Reynard et al. 2016) |

^a^ subscripts h=hair, w=water

**3. Background: Dietary reconstruction through carbon, nitrogen and sulphur isotope analysis of hair**

The stable carbon (δ^13^C), nitrogen (δ^15^N) and sulphur (δ ^34^S) isotope ratios of human and animal tissues largely reflect the δ^13^C, δ^15^N and δ^34^S values of ingested dietary protein (Ambrose and DeNiro 1987; DeNiro and Epstein 1978; DeNiro and Epstein 1981; Richards et al. 2003; Schoeninger and DeNiro 1984), with minor contributions of other macronutrients in the case of carbon (Froehle et al. 2010; Warinner and Tuross 2009). In archaeological studies, bone collagen is the most commonly selected analyte, and offers long-term (~10 years or more) dietary insights (Ambrose and Norr 1993; Hedges et al. 2007). However, where preserved, hair (keratin) offers a shorter-term record, growing at a rate of ~1cm per month in humans (Valkovic 1977) and thus offering the potential for time-series measurements across longer strands or locks (i.e., groups of strands) when they are cut into segments (serially-sampled) and analysed (Britton 2019; Britton et al. 2018b; Roy et al. 2005; Wilson et al. 2007). In purely C3 environments, such as northern Europe, carbon isotope ratios (δ^13^C) are most often used to discriminate between marine and terrestrial dietary protein sources, and nitrogen isotope ratios (δ^15^N) can be used to determine the trophic level of the protein consumed. In general, in pairwise comparisons of tissues, δ^15^N values increase by 3-5‰ with each step up the food chain, whereas δ^13^C values are enriched by ~1‰. δ^15^N values can also be used to indicate freshwater or marine dietary inputs, as aquatic ecosystems tend to have longer food chains (Richards et al. 2001). Due to the way in which dietary protein is metabolised and utilised in the body, there is a short delay of approximately 6 to 8 weeks before the isotope chemistry of growing hair reaches equilibrium with the diet (Huelsemann et al. 2009; Sponheimer et al. 2003). Therefore, the serial-sampling and measurement of δ^13^C and δ^15^N values in strands/locks of human hair can allow the identification of likely sources of human dietary protein, particularly the consumption of marine foods, during the period of time the hair grew minus a short time-lag.

Sulphur isotope ratios (δ^34^S) of hair also reflect dietary protein intake and can be used to distinguish between marine and terrestrial influences in the diet. Sulphur is also used as an indicator of geographical location during tissue growth, particularly coastal proximity (Nehlich 2015; Richards et al. 2003; Zazzo et al. 2011). Sulphur isotope ratios in plants and animals are derived from soil sulphur, which is influenced by both the local lithology and rainfall. Terrestrial organisms normally have sulphur isotope values of 5-10‰ (Peterson and Fry 1987) but can be closer to 0‰ (Nehlich and Richards 2009), and freshwater organisms exhibit similar values ranging from -5 (or lower) to 10-14‰ (Nehlich and Richards 2009; Nriagu et al. 1991). Oceanic sulphate has a mean value of ~20‰ (Rees et al. 1978), therefore marine organisms or organisms consuming marine protein are likely to have more elevated δ^34^S values closer to this value and at least above 14‰ (Nehlich and Richards 2009; Peterson and Fry 1987). However, oceanic sulphates alter soils and plants tens of kilometres inland, leading to elevated environmental δ^34^S values (the ‘sea spray effect’; Zazzo et al. 2011), meaning that coastal proximity can be one of the most dominant factors influencing isoscapes.

**4. Analytical protocols**

*4.1 Oxygen and hydrogen isotopes*

Oxygen and hydrogen isotopic determinations of hair were made at the Stable Isotope Laboratory in the Department of Geosciences at Boise State University, in Boise, Idaho, USA. Hair samples were equilibrated in a controlled fashion with local tap water (typically *δ*^2^H= ~ -125 ‰; *δ*^18^O= ~ -16.5 ‰, with little seasonal variation) to ensure all were treated equivalently. Segments of hair and samples of USGS 42 and USGS 43 hair reference materials were loosely wrapped in silver capsules and placed in a plastic 96-well tray without a lid. This tray was sealed in a large glass desiccator alongside a beaker of 50 ml of local tap water. Samples were equilibrated for 4 days in this atmosphere, then the tray was transferred to a plastic vacuum desiccator (Bel Art) connected to a vacuum line and exposed to vacuum for 7 days. Samples and USGS reference materials were then rapidly transferred to a Zero Blank autosampler (Costech) and purged with helium before mass spectrometric analysis.

Hydrogen and oxygen isotope values were measured with a Thermal Conversion Elemental Analyzer (TC/EA) coupled to a Delta Plus Isotope Ratio Mass Spectrometer (IRMS) with a Conflo IV interface (all from Thermo Scientific). The TC/EA was packed with a glassy carbon reactor and run at 1450 °C, and a 1.8 m GC column replaced the shorter factory-supplied one. The helium dilution in the sample flow path was increased to 89% with the Conflo IV after the H_2_ peak and before the CO peak, to reduce NO­_x_ species entering the IRMS source.

We used a glassy carbon packing for the TC/EA so that both hydrogen and oxygen isotope ratios could be obtained on the same hair segment, to maximize the data retrievable. With an alternative chromium-packed reactor no *δ*^18^O values can be obtained on the same sample aliquot since CO for *δ*^18^O determination is not formed in the presence of Cr. Hydrogen isotope values obtained with a glassy carbon-packed reactor are offset from those in a chromium-packed reactor due to formation of HCN in the former (see Gehre et al. 2015; Reynard et al. 2016; Reynard et al. 2019). This difference is approximately 12-14 ‰ for USGS 42 and 43 (δ^2^H values from a Cr-packed column are higher; Reynard and Tuross 2016), and 10.4 ± 1.1 (1 SD) ‰ in quail feathers (Reynard, unpublished data). The water reference materials VSMOW and SLAP in sealed silver tubes were measured in each run and used to drift correct, stretch, and normalize the data (Coplen 1988). The results are reported relative to VSMOW on the VSMOW-SLAP scale. We also included USGS 42 and USGS 43 (human hair reference materials) in each sample batch; these were treated equivalently to the hair segments and subject to the same water equilibration as described above. We also normalized the data using the recommended values of these materials (USGS 42: *δ*^2^H = -72.9 ± 2.2 ‰, *δ*^18^O = 8.56 ± 0.10 ‰; USGS 43: *δ*^2^H = -44.4 ± 2.0 ‰, *δ*^18^O= 14.11 ± 0.10 ‰). The hydrogen isotopic values given in the certificates for these USGS materials is that of the non-exchangeable hydrogen fraction measured with a Cr-packed reactor. We report both the USGS 42-USGS 43 normalized values and the VSMOW normalized values in SI Table S1. The USGS 42 and USGS 43 normalized values are comparable to those in Bataille et al. (2022), while the hair samples normalized to VSMOW with the standard waters directly are comparable to those in Reynard et al. (2016); the other studies in Table S1 are also reported relative to VSMOW but use a variety of keratinous and non-keratinous reference materials to make this normalization.

*4.2 Carbon, nitrogen and sulphur isotopes*

Stable nitrogen (δ^15^N), carbon (δ^13^C), and sulphur (δ^34^S) isotopic compositions were determined using a Delta V Advantage continuous-flow isotope ratio mass spectrometer coupled via a ConfloIV to an IsoLink elemental analyser (Thermo Scientific, Bremen) at The Scottish Universities Research Centre (SUERC), East Kilbride as described in Sayle et al. (2019). In a single reactor containing tungstic oxide and copper wires hair samples were combusted in the presence of oxygen at 1020 °C to produce N_2_, CO_2_ and SO_2_. A magnesium perchlorate trap was used to eliminate water produced during the combustion process, and the gases were separated in GC column heated between 70°C and 240°C. Helium was used as a carrier gas throughout the procedure. N_2_, CO_2_, and SO_2_ entered the mass spectrometer via an open split arrangement within the ConfloIV and were analysed against their corresponding reference gases. The International Atomic Energy Agency (IAEA) reference materials USGS40 (L-glutamic acid, δ^13^C_VPDB_ = –26.39 ± 0.04‰, δ^15^N_AIR_ = –4.52 ± 0.06‰) and USGS41a (L-glutamic acid, δ^13^C_VPDB_ = 36.55 ± 0.08‰, δ^15^N_AIR_ = 47.55 ± 0.15‰) were used to normalise δ^13^C and δ^15^N values. Two in-house standards (GS2, δ^34^S_VCDT_ = –10.28 ± 0.18‰ and GAS2, δ^34^S_VCDT_ = 18.56 ± 0.10‰) that are calibrated to the IAEA reference materials IAEA-S-2 (silver sulfide, δ^34^S_VCDT_ = 22.62 ± 0.08‰) and IAEA-S-3 (silver sulfide, δ^34^S_VCDT_ = –32.49 ± 0.08‰) were used to normalise δ^34^S values. Results are reported as per mil (‰) relative to the internationally accepted standards VPDB, AIR and VCDT. Normalisation was checked using the marine collagen USGS88 (^13^C_VPDB_ = –16.06 ± 0.07‰, δ^15^N_AIR_ = 14.96 ± 0.14‰, and δ^34^S_VCDT_ = 17.10 ± 0.44‰) and Indian human hair USGS43 (δ^13^C_VPDB_ = –21.28 ± 0.11‰, δ^15^N_AIR_ = 8.44 ± 0.10‰, and δ^34^S_VCDT_ = 10.46 ± 0.22‰).

**5. References**

Ambrose SH, and DeNiro MJ. 1987. Bone nitrogen isotope composition and climate. *Nature* 325:1987

Ambrose SH, and Norr L. 1993. Experimental evidence for the relationship of the carbon isotope ratios of whole diet and dietary protein to those of bone collagen and carbonate. In: Lambert JB, and Grupe G, editors. *Prehistoric Human Bone: Archaeology at the Molecular Level*. New York: Springer-Verlag. p 1-37.

Bataille CP, Ammer STM, Bhuiyan S, Chartrand MMG, St-Jean G, and Bowen GJ. 2022. Multi-isotopes in human hair: A tool to initiate cross-border collaboration in international cold-cases. *PLoS ONE* 17(10):e0275902.

Bowen GJ, Ehleringer JR, Chesson LA, Thompson AH, Podlesak DW, and Cerling TE. 2009. Dietary and physiological controls on the hydrogen and oxygen isotope ratios of hair from mid-20th century indigenous populations. *American Journal of Physical Anthropology* 139(4):494-504.

Britton K. 2019. New isotope evidence for diachronic and site-spatial variation in precontact diet during the Little Ice Age at Nunalleq, southwest Alaska. *ÉTUDES/INUIT/STUDIES* 43(1-2).

Britton K, McManus-Fry E, Cameron A, Duffy P, Masson-MacLean E, Czére O, Smith N, Stones J, Winfield A, and Müldner G. 2018a. Isotopes and new norms: Investigating the emergence of early modern U.K. breastfeeding practices at St. Nicholas Kirk, Aberdeen. *International Journal of Osteoarchaeology* 28:510– 522.

Britton K, McManus-Fry E, Nehlich O, Richards MP, Ledger PM, and Knecht RA. 2018b. Stable carbon, nitrogen and sulphur isotope analysis of permafrost preserved human hair from rescue excavations (2009, 2010) at the precontact site of Nunalleq, Alaska. *Journal of Archaeological Science: Reports* 17:950-963.

Coplen TB. 1988. Normalization of oxygen and hydrogen isotope data. *Chemical Geology: Isotope Geoscience Section* 72:293.

Czére O. 2020. From the Dark Ages to the Medieval State: A diachronic isotopic study of dietary change in Scotland, from the Early to the High Medieval Period. PhD Thesis: University of Aberdeen.

DeNiro MJ, and Epstein S. 1978. Influence of diet on the distribution of carbon isotopes in animals. *Geochimica et Cosmochimica Acta* 42:495-506.

DeNiro MJ, and Epstein S. 1981. Influence of diet on the distribution of nitrogen isotopes in animals. *Geochimica et Cosmochimica Acta* 45:341-351.

Duffy P, Arabaolaza I, and Kilpatrick M. 2008. Draft report: The human remains from the St. Nicholas Uniting, Aberdeen. Unpublished GUARD osteoarchaeological report.

Ehleringer JR, Bowen GJ, Chesson LA, West AG, Podlesak DW, and Cerling TE. 2008. Hydrogen and oxygen isotope ratios in human hair are related to geography. *Proceedings of the National Academy of Sciences of the United States of America* 105(8):2788-2793.

Fraser I, and Meier-Augenstein W. 2007. Stable 2H isotope analysis of modern-day human hair and nails can aid forensic human identification. *Rapid Communications in Mass Spectrometry* 21(20):3279-3285.

Froehle AW, Kellner CM, and Schoeninger MJ. 2010. FOCUS: effect of diet and protein source on carbon stable isotope ratios in collagen: follow up to Warinner and Tuross (2009). *Journal Of Archaeological Science* 37:2662-2670.

Gehre M, Renpenning J, Gilevska T, Qi H, Coplen TB, Meijer HA, Brand WA, and Schimmelmann A. 2015. On-line hydrogen-isotope measurements of organic samples using elemental chromium: an extension for high temperature elemental-analyzer techniques. *Analytical Chemistry* 87(10):5198-5205.

Hedges REM, Clement JG, Thomas CDL, and O'Connell TC. 2007. Collagen turnover in the adult femoral mid-shaft: modeled from anthropogenic radiocarbon tracer measurements. *American Journal of Physical Anthropology* 133:808-816.

Hobson KA, and Wassenaar LI. 2008. Tracking animal migration with stable isotopes. Amsterdam: Academic Press. xi, 144 p., [144] p. of plates p.

Huelsemann F, Flenker U, Koehler K, and Schaenzer W. 2009. Effect of a controlled dietary change on carbon and nitrogen stable isotope ratios of human hair. *Rapid Communications in Mass Spectrometry* 23(16):2448-2454.

Hunter J. 1972. The Church of St. Nicholas, Aberdeen. *Proceedings of the Society of Antiquaries of Scotland* 105:236-247.

Lehn C, Rossmann A, and Graw M. 2015. Provenancing of unidentified corpses by stable isotope techniques–presentation of case studies. *Science and Justice* 55(1):72-88.

Nehlich O. 2015. The application of sulphur isotope analyses in archaeological research: A review. *Earth-Science Reviews* 142:1-17.

Nehlich O, and Richards MP. 2009. Establishing collagen quality criteria for sulphur isotope analysis of archaeological bone collagen. *Archaeological and Anthropological Sciences*.

Nriagu J, Rees CE, Mekhtieva VL, Lein AY, Fritz P, Drimmie RJ, Pankina RG, Robinson RW, and Krouse HR. 1991. Sulfur Isotopes in the Hydrosphere. In: Krouse HR, and Grinenko VA, editors. *Stable Isotopes: Natural and Anthropogenic Sulphur in the Environment*. Chichester: John Wiley & Sons. p 177-265.

O'Brien DM, and Wooller MJ. 2007. Tracking human travel using stable oxygen and hydrogen isotope analyses of hair and urine. *Rapid Communications in Mass Spectrometry* 21(15):2422-2430.

Oram R. 2016. The Medieval Church in the Dioceses of Aberdeen and Moray. In: Geddes J, editor. *Medieval Art, Architecture and Archaeology in the Dioceses of Aberdeen and Moray*. Leeds: Routledge. p 16-32.

Peterson BJ, and Fry B. 1987. Stable isotopes in ecosystem studies. *Annual Review of Ecology and Systematics* 18:293-320.

Rees CE, Jenkins WJ, and Monster J. 1978. The sulphur isotopic composition of ocean water sulphate. *Geochimica et Cosmochimica Acta* 42:377-381.

Reynard LM, Burt N, Koon HEC, and Tuross N. 2016. Limits and possibilities in the geolocation of humans using multiple isotope ratios (H, O, N, C) of hair from east coast cities of the USA. *Isotopes in Environmental and Health Studies* 52(4-5):498-512.

Reynard LM, Ryan SE, and Tuross N. 2019. The interconversion of δ2H values of collagen between thermal conversion reactor configurations. *Rapid Communications in Mass Spectrometry* 33(7):678-682.

Reynard LM, and Tuross N. 2016. Hydrogen isotopic analysis with a chromium‐packed reactor of organic compounds of relevance to ecological, archaeological, and forensic applications. *Rapid Communications in Mass Spectrometry* 30(16):1857-1864.

Richards MP, Fuller BT, Sponheimer M, Robinson T, and Ayliffe L. 2003. Sulphur isotopes in palaeodietary studies: a review and results from a controlled feeding experiment. *International Journal of Osteoarchaeology* 13:37-45.

Richards MP, Pettitt PB, Stiner MC, and Trinkaus E. 2001. Stable isotope evidence for increasing dietary breadth in the European mid-Upper Paleolithic. *Proceedings of the National Academy of Sciences of the United States of America* 98(11):6528-6532.

Roy DM, Hall R, Mix AC, and Bonnichsen R. 2005. Using Stable Isotope Analysis to Obtain Dietary Profiles from Old Hair: A Case Study from Plains Indians. *American Journal of Physical Anthropology* 128:444-452.

Sayle KL, Brodie CR, Cook GT, and Hamilton WD. 2019. Sequential measurement of δ^15^N, δ^13^C and δ^34^S values in archaeological bone collagen at the Scottish Universities Environmental Research Centre (SUERC): A new analytical frontier. *Rapid Communications in Mass Spectrometry* 33(15):1258-1266.

Schoeninger MJ, and DeNiro MJ. 1984. Nitrogen and carbon isotopic composition of bone collagen from marine and terrestrial animals. *Geochimica et Cosmochimica Acta* 48:625-639.

Sharp Z. 2017. Principles of stable isotope geochemistry: University of New Mexico.

Sharp ZD, Atudorei V, Panarello HO, Fernández J, and Douthitt C. 2003. Hydrogen isotope systematics of hair: archeological and forensic applications. *Journal Of Archaeological Science* 30(12):1709-1716.

Sponheimer M, Robinson T, Ayliffe L, Roeder B, Hammer J, Passey B, West A, Cerling T, Dearing D, and Ehleringer J. 2003. Nitrogen Isotopes in Mammalian Herbivores: Hair δ^15^N Values from a Controlled Feeding Study. *International Journal of Osteoarchaeology* 13:80-87.

Thompson AH, Chesson LA, Podlesak DW, Bowen GJ, Cerling TE, and Ehleringer JR. 2010. Stable isotope analysis of modern human hair collected from Asia (China, India, Mongolia, and Pakistan). *American Journal of Physical Anthropology* 141(3):440-451.

Valkovic V. 1977. Trace Elements in Human Hair. New York: Garland STPM Press.

Warinner C, and Tuross N. 2009. Alkaline cooking and stable isotope tissue-diet spacing in swine: archaeological implications. *Journal Of Archaeological Science* 36:1690-1697.

Wilson AS, Taylor T, Ceruti MC, Chavez JA, Reinhard J, Grimes V, Meier-Augenstein W, Cartmell L, Stern B, Richards MP, Worobey M, Barnes I, and Gilbert MTP. 2007. Stable isotope and DNA evidence for ritual sequences in Inca child sacrifice. *Proceedings of the National Academy of Sciences of the United States of America* 104(42):16456-16461.

Zazzo A, Monahan FJ, Moloney AP, Green S, and Schmidt O. 2011. Sulphur isotopes in animal hair track distance to sea. *Rapid Communications in Mass Spectrometry* 25(17):2371-2378.

**6. SI Appendix**

(Blank) copy of short questionnaire from modern participant study.

**
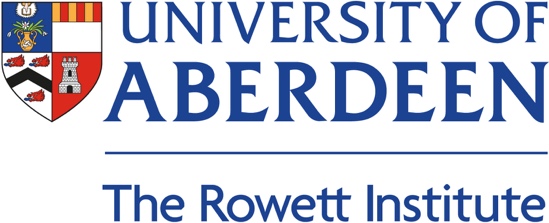
**

**Short Questionnaire**

**Study: Measuring vitamin D in hair samples – a method development study**

**Participant number: ……………………………….**

**Date: …………………………………………**

**1. Gender:** Female ☐ Male ☐ Other ☐ Prefer not to answer ☐

**2. Please specify how you would describe your ethnic origin:**

☐ White -British ☐ Asian or Asian British Bangladeshi

☐ White Irish ☐ Chinese

☐ White Scottish ☐ Other Asian Background

☐ Irish Traveller ☐ Mixed White and Black Caribbean

☐ Other White Background ☐ Mixed White and Black African

☐ Black or Black British Caribbean ☐ Mixed White and Asian

☐ Black or Black British African ☐ Other Mixed backgrounds

☐ Other Black background ☐ Other Ethnic background

☐ Asian or Asian British Indian ☐ Asian or Asian British Pakistani

☐ Arab ☐ Not Known

☐ Prefer not to disclose

**3. What is your age? ……………**

**4. What is your height? ………………..**

**5. What is your weight? ………………..**

**6. What is the original colour of your hair?**

☐ Black ☐ Blond ☐ Brown ☐ Red

**7. Do you currently take daily vitamin D supplements, or multivitamins containing vitamin D?**

Yes ☐ No ☐

**8. Do you currently take any other foods or supplements containing vitamin D?**

Yes ☐ No ☐ Don’t know ☐

**9. How often do you eat oily fish (salmon, mackerel, herring)?**

More than once a week ☐

2-4 times a month ☐

Once a month ☐

Hardly or never ☐

**10. On average, how many minutes or hours approximately do you spend outside on a day?**

**…………………………………………..**

**11. Have you lived in the North East of Scotland for the past three years?**

Yes ☐ No ☐

**12. Have you chemically treated your hair (dyed, permed, bleached or other treatment)?**

Yes ☐ No ☐
